# FW-04-806 inhibits proliferation and induces apoptosis in human breast cancer cells by binding to N-terminus of Hsp90 and disrupting Hsp90-Cdc37 complex formation

**DOI:** 10.1186/1476-4598-13-150

**Published:** 2014-06-14

**Authors:** Wei Huang, Min Ye, Lian-ru Zhang, Qun-dan Wu, Min Zhang, Jian-hua Xu, Wei Zheng

**Affiliations:** 1 School of Pharmacy, Fujian Medical University, Basic Medicine Building North 205, No.88 Jiaotong Road, Fuzhou, Fujian 350004, China; 2 Fujian Institute of Microbiology, Fuzhou 350007, China; 3 Fuijan Provincial Key Laboratory of Natural Medicine Pharmacology, Fuzhou 350004, China; 4 School of life Sciences, Xiamen University, Xiamen 361005, China

**Keywords:** FW-04-806, Hsp90, Cdc37, HER2, Breast cancer

## Abstract

**Background:**

Heat shock protein 90 (Hsp90) is a promising therapeutic target and inhibition of Hsp90 will presumably result in suppression of multiple signaling pathways. FW-04-806, a bis-oxazolyl macrolide compound extracted from China-native *Streptomyces* FIM-04-806, was reported to be identical in structure to the polyketide Conglobatin.

**Methods:**

We adopted the methods of chemproteomics, computational docking, immunoprecipitation, siRNA gene knock down, Quantitative Real-time PCR and xenograft models on the research of FW-04-806 antitumor mechanism, through the HER2-overexpressing breast cancer SKBR3 and HER2-underexpressing breast cancer MCF-7 cell line.

**Results:**

We have verified the direct binding of FW-04-806 to the N-terminal domain of Hsp90 and found that FW-04-806 inhibits Hsp90/cell division cycle protein 37 (Cdc37) chaperone/co-chaperone interactions, but does not affect ATP-binding capability of Hsp90, thereby leading to the degradation of multiple Hsp90 client proteins via the proteasome pathway. In breast cancer cell lines, FW-04-806 inhibits cell proliferation, caused G2/M cell cycle arrest, induced apoptosis, and downregulated Hsp90 client proteins HER2, Akt, Raf-1 and their phosphorylated forms (p-HER2, p-Akt) in a dose and time-dependent manner. Importantly, FW-04-806 displays a better anti-tumor effect in HER2-overexpressed SKBR3 tumor xenograft model than in HER2-underexpressed MCF-7 model. The result is consistent with cell proliferation assay and *in vitro* apoptosis assay applied for SKBR-3 and MCF-7. Furthermore, FW-04-806 has a favorable toxicity profile.

**Conclusions:**

As a novel Hsp90 inhibitor, FW-04-806 binds to the N-terminal of Hsp90 and inhibits Hsp90/Cdc37 interaction, resulting in the disassociation of Hsp90/Cdc37/client complexes and the degradation of Hsp90 client proteins. FW-04-806 displays promising antitumor activity against breast cancer cells both *in vitro* and *in vivo*, especially for HER2-overexpressed breast cancer cells.

## Introduction

Heat shock protein (Hsp) 90 is a highly conserved chaperone protein and among the most abundant proteins found in eukaryotic cells [1-3]. Hsp90 exists as a homodimeric structure in which individual monomers are each characterized by three domains: an N-terminal nucleotide binding domain (NBD), the site of ATP binding; the middle domain (MD), involved in ATP hydrolysis and the site of co-chaperone and client protein binding; and a C-terminal dimerization domain (CDD), the site of dimerization [4]. In addition to protecting cells by correcting misfolded proteins, Hsp90 also plays a key role in regulating the stability, maturation, and activation of a wide range of client substrates, including kinases, hormone receptors, and transcription factors [5-8]. Most Hsp90 client proteins, such as epidermal growth factor receptor 2 (HER2), Akt, Raf-1, Cdk4, Bcr-Abl, and p53, are essential for cancer cell survival and proliferation [9]. The chaperoning of these client proteins is regulated by a dynamic cycle driven by ATP binding to Hsp90 and subsequent hydrolysis of the protein [10]. Hsp90 requires a series of co-chaperones to form a complex in order to function. These co-chaperones, including cell division cycle protein 37 (Cdc37), Hsp70, Hsp40, Hop, Hip, p23, pp5, and immunophilins, bind to the super-chaperone complex and are released at various time points to regulate the folding, assembly, and maturation of Hsp90 client proteins [11]. To date, the mechanisms of developed Hsp90 inhibitors have greatly expanded, ranging from the Hsp90 protein function inhibitor to agents targeting the function of nucleotides and co-chaperones crucially involved in regulating the Hsp90 cycle [4].

We adopted chemoproteomics-based drug screening [12,13] to identify clinical Hsp90 inhibitor candidates among a series of natural product, extracted from plants, Fungus, actinomycetes secondary metabolites and so on. Specifically, the histidine-tagged yeast Hsp90 was loaded onto an affinity column [12] and was subsequently tested with these natural product. Mass spectrum analysis of the eluted solutions of proteins resulted in the identification of the compounds bound to Hsp90. This primary screening effort led to the discovery of FW-04-806 as one of the potential Hsp90 inhibitors. Secondary screening was conducted in parallel across multiple targets. The FW-04-806-loaded affinity columns were incubated with the histidine-tagged NBD, MD, and CDD of yeast Hsp90 to provide substantial binding information and relative binding affinities.

FW-04-806, extracted from the China-native *Streptomyces* FIM-04-806 [14], is identical in structure to Conglobatin [15] according to ultraviolet spectra, infrared spectra, and NMR (^1^H and ^13^C) data and single-crystal X-ray diffraction data [16]. Cell proliferation assays have shown that FW-04-806 inhibits the growth of a human chronic myelocytic leukemia K562 cell line with an IC_50_ of 6.66 μg/mL (almost 10 μM) [16]. Conglobatin has been reported to be non-toxic at doses up to 1 g/kg when administered to mice either orally or interperitoneally [15]. In addition, our acute toxicity test showed that mice survived after oral administration of 900 mg/kg of FW-04-806. In the present study, we investigated the effects of FW-04-806 on SKBR3 and MCF-7, HER2-overexpressed and HER2-underexpressed human breast cancer cell lines, respectively. Chemoproteomics and computational approaches together confirmed that FW-04-806 bound to the N-terminus of Hsp90. A colorimetric assay for inorganic phosphates and ATP pull-down assay showed that FW-04-806 had little effect on Hsp90 ATPase activity compared with 17AAG and did not affect ATP-binding of Hsp90. Indeed, immunoprecipitation confirmed that FW-04-806 disrupted Hsp90/Cdc37 chaperone/co-chaperone interactions, leading to enhanced tumor-arresting activity--and caused the degradation of Hsp90 client proteins. In addition, FW-04-806 exhibited anticancer activity in an *in vivo* breast cancer xenograft model, and no major toxicity was observed in the animals. These data suggest that FW-04-806 is a potent Hsp90 inhibitor against human breast cancer cells.

## Materials and methods

### Cell lines and reagents

SKBR3 and MCF-7 breast cancer cells were originally obtained from American type culture collection. SKBR3 cells were cultured in Roswell Memorial Park Institute-1640 medium and MCF-7 cells were grown in Dulbecco’s modified Eagle medium. All media were supplemented with 10% fetal bovine serum. The cells were maintained under standard cell culture conditions at 37°C and 5% CO_2_ in a humid environment.

FW-04-806 (purity ≥98.5%) was produced by Fujian Institute of Microbiology, China [14,16]. Recombinant human Cdc37 was obtained from Sino Biological Inc. MG132 was obtained from Sigma Aldrich. 17AAG (Tanespimycin) was purchased from Selleckchem. MTS was obtained from Promega. Primary antibodies against Hsp90, Neu, Akt, Raf-1, His-probe and β-actin were purchased from Santa Cruz Biotechnology. Primary antibodies against phospho-Akt (Thr308), apoptosis and phospho-HER2/ErbB2 antibody sampler kits containing cleaved caspase-3 (Asp175), caspase-3, poly (ADP-ribose) polymerase (PARP), cleaved PARP (Asp214), caspase-9, cleaved caspase-9 (Asp330), caspase-7, cleaved caspase-7 (Asp 198), HER2/ErbB2 (D8F12), and phospho-HER2/ErbB2 (Tyr1221/1222) were obtained from Cell Signaling Technology. An Annexin V: fluorescein isothiocyanate (FITC) Apoptosis Detection Kit I was purchased from BD Biosciences.

### Preparation of Hsp90 protein

Recombinant vectors were constructed for histidine-tagged full-length (1–732, 90 kDa), NBD (1–236, 25 kDa), MD (272–617, 40 kDa), and CDD (629–732, 15 KDa) of yeast Hsp90. The fusion proteins were expressed in BL21(DE3) and purified via Ni-NTA column and gel filtration [17].

### Resin synthesis

CNBr-activated Sepharose™4B (GE Healthcare) was swelled in 1 mM HCl and washed with coupling buffer (0.1 M NaHCO_3_, 0.5 M NaCl, pH = 8.3).

For the Hsp90-loaded affinity column, 10 mg of protein per mL of medium was added to the resin, the mixture was rotated overnight at 4°C, and then washed with coupling buffer. Any remaining active groups were blocked with capping solution (1 M ethanolamine) at room temperature for 2 h. The resin was then treated with a small molecule compound, rotated end to overend at room temperature for 4 h, and then washed away of any excess compound. The resin was then washed with three rounds of high pH buffer (0.1 M Tris–HCl, 0.5 M NaCl, pH = 8)/low pH buffer (0.1 M AcOH/NaAcOH, 0.5 M NaCl, pH = 4). Samples were desalted using a Vivapure C18 spin column (Sartorius) before LC-MS analysis [18].

For the drug-loaded affinity column, after the resin was swelled, washed, and added into coupling buffer, FW-04-806 was dissolved in dimethyl sulfoxide (DMSO) and mixed into the resin (up to 10 μmoles per mL of medium). The mixture was rotated end to overend for 4 h at room temperature, and then washed away of the excess ligand with coupling buffer. Any remaining active groups were blocked with the capping solution for 2 h at room temperature, and the column was equilibrated with coupling buffer. The test proteins were added into the resin, the mixture was rotated overnight at 4°C, and then washed away of any excess proteins. The resin was added to loading buffer, boiled for 10 min, separated with 10% sodium dodecyl sulfate polyacrylamide gel electrophoresis, and then assayed by western blotting.

### LC-MS detection

Samples were analyzed on Agilent 6410B Triple Quadrupole LC/MS system. Peptides were separated on a BioBasic Picofrit C18 capillary column (New Objective). Elution was performed with an acetonitrile gradient from 0 to 100% over 1 h with an overall flow rate of 1 mL/min.

### ATP-Sepharose binding assay

ATP-Sepharose binding assay was modified base on previous protocol [19]. Different concentrations of FW-04-806 or 17AAG were added into recombinant NBD Hsp90 protein (10 μg), and then mixtures were incubated with 25 µL preequilibrated γ-phosphate-linked ATP-Sepharose (Jena Bioscience GmbH) in 200 µL incubation buffer (10 mM Tris–HCl, 50 mM KCl, 5 mM MgCl_2_, 20 mM Na_2_MoO_4_ , 0.01% NP-40, pH 7.5) for 4 h at 4°C. The protein bound to Sepharose beads was separated with 10% sodium dodecyl sulfate polyacrylamide gel electrophoresis and assayed with protein immunoblotting.

### Colorimetric determination of ATPase activity

Malachite green reagent [20,21] was prepared on the day of use and contained malachite green (0.0812%, w/v), polyvinyl alcohol (2.32%, w/v, dissolves with difficulty and requires heating), ammonium molybdate (5.72%, w/v, in 6 M HCl), and argon water mixed in a ratio of 2:1:1:2 to a golden yellow solution. The assay buffer consisted of 100 mM Tris–HCl, 20 mM KCl, and 6 mM MgCl_2_, with a pH of 7.4. The experiments were performed in 100 μL of test solution containing 80 μL of malachite green reagent. The test solution contained 0.5 μM Hsp90 protein, 1 mM ATP, and 25, 50, 100, or 200 μM FW-04-806 or vehicle (DMSO).

### Immunoprecipitation

Samples (500 μg of total protein) were incubated overnight with 2 μg of primary antibody at 4°C, after which 20 μL of protein A Mag Sepharose™ (GE Healthcare, UK) was added to the mixture, which was then incubated for 2 h at 4°C. The immunoprecipitated protein complexes were washed once with lysis buffer and twice with ice-cold PBS. After the supernatant was discarded, the antibody/protein complexes were resuspended in 30 μL of loading buffer and boiled for 5 min. The entire sample was separated with 10% sodium dodecyl sulfate polyacrylamide gel electrophoresis and assayed with protein immunoblotting.

### Small interfering RNA (siRNA) gene knockdown

SKBR3 cells were seeded in antibiotic-free normal growth medium supplemented with fetal bovine serum. Single siRNA oligonucleotides targeting human Hsp90α/β (sc-35608, Santa Cruz Biotechnology) and control siRNA (sc-37007) were diluted in siRNA Transfection Medium (sc-36868) and mixed with siRNA Transfection Reagent (sc-29528) according to the manufacturer’s protocol. SKBR3 cells were incubated with the transfection complexes for 6 h and in the normal growth medium for 48 h. Cells then were treated with DMSO or FW-04-806 for 24 h before cell lysates were prepared and analyzed with western blot.

### Quantitative real-time PCR

Total RNA extraction was performed with TRIzol reagent (Life Technologies Corporation), and first strand cDNA was synthesized using 1 μg of total RNA (concentrations measured by NANODROP 2000, Thermo Scientific) treated with avian myeloblastosis virus (AMV) reverse transcriptase (Promega) according to the manufacturer’s instructions. Quantitative real-time reverse transcription polymerase chain reaction (RT-PCR) analysis was performed in triplicate with FastStart Essential DNA Green Master (Roche) using LightCycler 96 (Roche). The ΔΔCT method was used to calculate relative expression. Primer sequences used in RT-PCR for human Akt (forward 5′-TTGAGAGAAGCCACGCTGT-3′ and reverse 5′-CGGAGAACAAACTGGATGAA-3′), HER2 (forward 5′-TGCTGTCCTGTTCACCACTC-3′ and reverse 5′-TGCTTTGCCACCATTCATTA-3′), Raf-1 (forward 5′-CACCTCCAGTCCCTCATCTG-3′ and reverse 5′-CTCAATCATCCTGCTGCTCA-3′), Hsp90 (forward 5′-GGGCAACACCTCTACAAGGA-3′ and reverse 5′-ATCAACTGGGCAATTTCTGC-3′), GAPDH (forward 5′-AGAAGGCTGGGGCTCATTTG-3′ and reverse 5′-AGGGGCCATCCACAGTCTTC-3′).

### MTS assay

Cells (5 × 10^3^/well) were seeded into 96-well plates and treated with 10, 20, or 40 μM of FW-04-806 or vehicle (DMSO) for 48 h. At the end of the incubation period, cell viability was assessed by MTS assay(Promega)according to the manufacturer’s instruction. The number of living cells is proportional to the absorbance at 490 nm. The results are presented as means ± standard deviation from three independent experiments. Inhibition graphs used mean values obtained from each concentration relative to control values, and the half maximal inhibitory concentration (IC_50_) were calculated by SPSS.

### Cell cycle analysis

Cells were seeded in 6-well plates and treated with various doses of FW-04-806 or vehicle (DMSO) for 24 h. The cells were harvested, washed with phosphate-buffered saline (PBS), and fixed with 70% ethanol at −20°C overnight. After an additional washing, cells were incubated with RNase A (20 μg/mL) at 37°C for 30 min, stained with propidium iodide (100 μg/mL; Sigma Aldrich) for 10 min, and analyzed with flow cytometry (BD FACSC autoTM II).

### Apoptosis assay

Apoptosis was determined with the Annexin-V: FITC Apoptosis Detection Kit I (BD Biosciences) according to the manufacturer’s protocol. Briefly, the vehicle control (DMSO) and FW-04-806-treated cells were collected via centrifugation and washed once with PBS. The cells were subsequently stained with fluorescein and propidium iodide for 15 min at room temperature and analyzed with flow cytometry.

### Western blot analysis

After treatment, cancer cells were collected, washed with PBS, lysed with NP-40 lysis buffer (50 mmol/L Tris pH 8.0, 150 mmol/L NaCl, and 1% NP-40) supplemented with phenylmethanesulfonyl fluoride (Sigma Aldrich) and PhosSTOP (Roche Diagnostics) for 30 min at 4°C, and centrifuged at 12,000 × *g* for 10 min. The supernatant was collected as the total protein extract. Protein concentration was estimated using a Pierce BCA Protein Assay Kit (Thermo Scientific, USA) according to the manufacturer’s protocol. Equal amounts of protein were analyzed with sodium dodecyl sulfate polyacrylamide gel electrophoresis. Thereafter, proteins were transferred to polyvinylidene fluoride membranes and blotted with specific primary antibodies. Proteins were detected via incubation with horseradish peroxidase-conjugated secondary antibodies and visualized with SuperSignal WestPico (Thermo Scientific, USA). All the western blot detections were repeated three or more times.

### Animals, tumor xenografts, and test agents for *in vivo* studies and efficacy

BALB/c (nu/nu) athymic mice were purchased from Shanghai SLAC Laboratory Animal Co. LTD. For SKBR3 and MCF-7 xenografts, 6-mm^3^ tumor fragments were implanted into the subcutaneous tissue of the axillary region using a trocar needle, and the animals were randomly divided into groups (n = 6) when the bearing tumor reached approximately 20 mm^3^. FW-04-806 was suspended at the desired concentration for each dose group in an aqueous vehicle containing 10% ethanol, 10% polyethylene glycol 400, and 10% Tween 80. The control group was given 0.4 mL/mouse vehicle solution i.g.; mice in other groups were given 50, 100, or 200 mg/kg of FW-04-806. Doxorubicin hydrochloride (ADM, Shenzhen Main Luck Pharmaceuticals Inc, China) was purchased as 10 mg injections and diluted with saline as necessary to achieve the prescribed concentration.

Tumor volumes were calculated using the following ellipsoid formula: [D × (d^2^)]/2, in which D is the large diameter of the tumor, and d is the small diameter. Tumor growth inhibition was determined using the following formula: 100 % × [(WC–WT)/WC], in which WC represents mean tumor weight of a vehicle group, and WT represents mean tumor weight of a treated group. All animal experiments were approved by animal care and use committee, Fujian Medical University, China.

### Immunohistochemistry

Immunohistochemistry was performed on tumors harvested from each xenograft group treated with FW-04-806 or vehicle. Tumors were cut into 3- to 5-mm pieces, fixed in 4% paraformaldehyde for 6 h, dehydrated, paraffin-imbedded, sectioned, and placed on slides (Zhongshan Biotechnology Company, China). Antigen retrieval was performed in 0.1 M citrate buffer, pH 6, at 100°C for 2 min. After incubation with 3% hydrogen peroxide for 10 min and three washes with PBS buffer, primary antibody Neu (rabbit poly-clonal, Santa Cruz Biotechnology, sc-284) was used at a dilution of 1:200 for 2 h. The slides were sequentially incubated with biotin-conjugated secondary antibodies (1:200) followed by horseradish peroxidase-conjugated streptavidin (1:100). The first step lasted 20 min and the second step lasted 30 min at 37°C, with 5 min PBS washes three times for each step. The reactions were revealed using diaminobenzidine substrate, and the slides were then washed under running tap water. Contrast was applied with hematoxylin, and the slides were mounted in Canadian balsam and observed with a light microscope.

### Statistical analysis

Analysis of variance was used for comparisons across multiple groups. The data are reported as means ± standard deviation. Statistical analysis was conducted using PASWstatistics 18 (SPSS, Inc); p < 0.05 was considered statistically significant.

## Results

### FW-04-806 binds to the N-terminal of Hsp90

**Figure 1:**
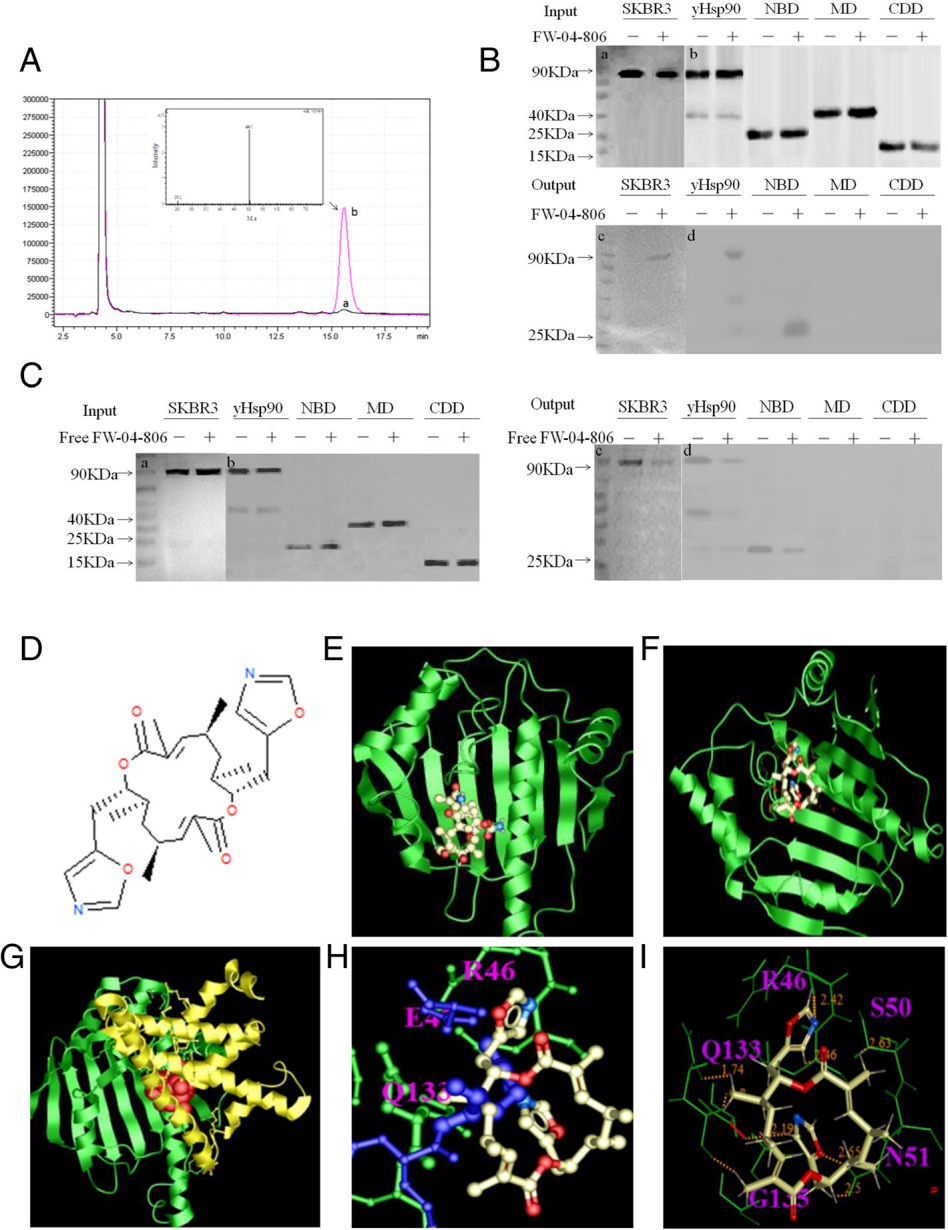
**FW-04-806 is identified as an Hsp90 binding medicine. (A)** LC/MS spectrum detects the compound eluted from the Hsp90-loaded affinity column. Peak a and b represent elution samples from the control and FW-04-806 bound Hsp90-loaded column, respectively. MS spectrum displays M/z of Peak b. **(B)** Western blot confirms FW-04-806 binds to NBD of Hsp90. The “–” symbol represents no drug-loaded affinity column, the “+” symbol represents the drug-loaded affinity column. The test proteins are SKBR3 cell lysate, His-tagged yeast Hsp90, and His-tagged NBD, MD, CDD of yeast Hsp90. Human Hsp90 Antibody and His-probe antibody were used respectively. **(C)** Soluble FW-04-806 was added into SKBR3 cell lysate, His-tagged full-length, NBD, MD and CDD of yeast Hsp90 up to 10 μM before incubation with drug-loaded affinity resin. Picture a and c show Human Hsp90 Antibody; picture b and d show His-probe antibody. **(D)** Molecular structure of FW-04-806. **(E)** Geldanamycin (GA) binds to N-terminal Hsp90 (Protein Data Bank [PDB] ID 1YTE) in the ATP binding pocket. Hsp90 is shown in the green ribbon view; GA is shown in stick view. **(F)** FW-04-806 docks to N-terminal Hsp90 (PDB ID 2K5B). N-Hsp90 is shown in green ribbon view; FW-04-806 is shown in stick view. **(G)** FW-04-806 binds to the N-Hsp90/Cdc37 complex (PDB ID 1US7). N-Hsp90 is shown in green ribbon view; Cdc37 is shown in yellow ribbon view; FW-04-806 is shown in orange ball view. **(H)** Stick view of FW-04-806 bound to N-Hsp90/Cdc37 complex. N-Hsp90 is shown in green stick view; FW-04-806 is shown in white stick view; Cdc37 is shown in blue stick view. **(I)** Stick views of FW-04-806 and N-Hsp90 in white and green, respectively.

FW-04-806, extracted from the China-native *Streptomyces* FIM-04-806 and identical to Conglobatin [16], had been discovered to be one of the potential Hsp90 inhibitors in the initial screening (Figure 1A). Secondly, the FW-04-806-loaded affinity columns were separately incubated with the histidine-tagged full-length, NBD, MD, and CDD of yeast Hsp90. This affinity-based screen showed that FW-04-806 bound to NBD of Hsp90, but not MD or CDD (Figure 1B). To define physiologically relevant associations with Hsp90, we added free soluble FW-04-806 up to 10 μM into proteins prior to exposure to the drug-loaded affinity resin [12]. The competition results showed that the soluble FW-04-806 can compete the binding of Hsp90 protein from cell lysate, full-length and NBD recombinant protein to the resin compared with the no free ligand adding control, which confirmed the specific interaction between NBD Hsp90 and FW-04-806 (Figure 1C).We then used Molsoft ICM 3.5a to model the interaction between Hsp90 (Protein Data Bank ID 2CCS) and FW-04-806 (Figure 1D). In contrast to the binding of GA with N-terminal Hsp90 (Figure 1E), FW-04-806 docked to different sites of N-terminal Hsp90, close to helix 4 or 5 (Figure 1F). Compared with the Hsp90/Cdc37 complex (PDB ID 2K5B), FW-04-806 bound to similar sites with the co-chaperone Cdc37 (Figure 1G).Electrostatic interactions form between the charge group of FW-04-806 and the amino group of residue R46/E47 of Hsp90 (Figure 1H). Hydrogen bonds are also formed between FW-04-806 and residues S50 and N51 in Hsp90 (Figure 1I). Specifically, hydrophobic packing interactions form between residue Q133 and the hydrophobic parts of FW-04-806 (Figure 1H). Of particular interest, FW-04-806 and Cdc37 share common binding sites at residues Q133 and E47/R46 of Hsp90 (Figure 1H).

The results of the chemoproteomics screening and docking models provide evidence that FW-04-806 is a potential inhibitor of Hsp90 by binding to the NBD of Hsp90.

### FW-04-806 does not affect ATP-binding capability of Hsp90, but inhibits Hsp90/Cdc37 chaperone/co-chaperone interactions

**Figure 2:**
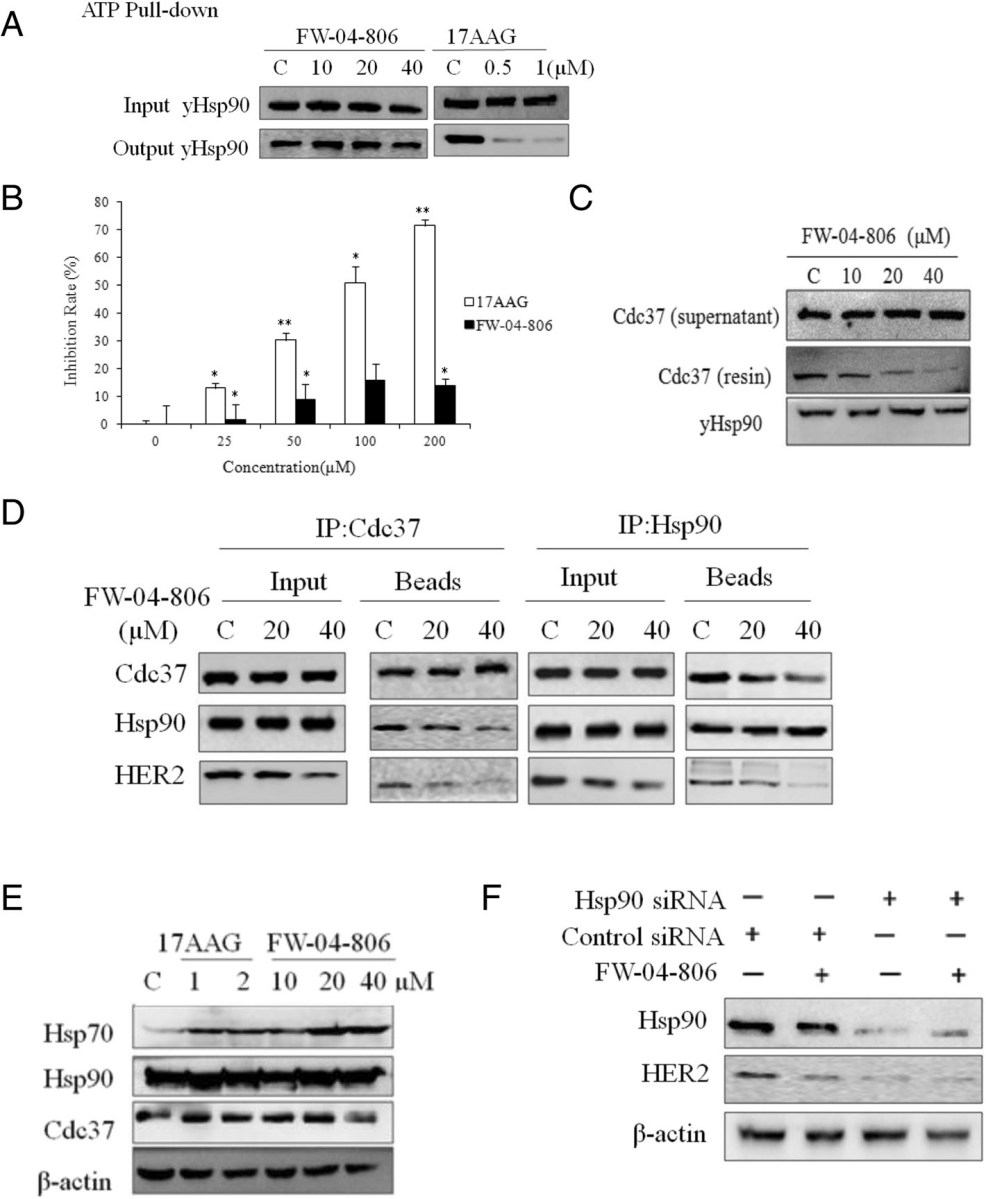
**FW-04-806 inhibits Hsp90/Cdc37 chaperone/co-chaperone interactions. (A)** FW-04-806 (10, 20, 40 μM) was added into recombinant NBD Hsp90 protein did not affect ATP binding capacity of Hsp90, while 17AAG (0.5, 1 μM) decreased ATP binding. Western blot was analyzed with His-probe antibody. **(B)** FW-04-806 had little effect on Hsp90 ATPase activity as determined by the malachite green reagent. The assay used 0.5 μM Hsp90 protein, 1 mM ATP, and FW-04-806 or 17AAG at 25, 50, 100, or 200 μM, or vehicle (DMSO) at 620 nm. Results are presented as means ± SD of three independent experiments. *p < 0.05: significant difference from control by analysis of variance; **p < 0.01: very significant difference from control by analysis of variance. **(C)** FW-04-806 directly affect Hsp90-Cdc37 interaction in Pull-down assay. 400 ug of purified Hsp90 (His-tag)protein was bound with Ni-NTA resin and divided into four groups evenly with the addition of recombinant Cdc37 protein 50 μg each and FW-04-806 0, 10, 20, 40 μM, respectively. After incubation and wash, The resins were boiled with loading buffer and analyzed by western blotting, His-probe antibody and Cdc37 antibody was used respectively. **(D)** SKBR3 cells were treated with FW-04-806 or DMSO for 24 h. Cdc37 or Hsp90 were immunoprecipitated from whole-cell lysates (500 μg each) with an anti-Cdc37 or anti-Hsp90 antibody respectively, then analyzed by immunoblotting with antibody against Hsp90, Cdc37 and HER2 **(E)** SKBR3 cells were treated with FW-04-806 at 10, 20, 40 μM for 24 h; 17AAG was used as a positive control at 1 and 2 μM. Hsp70, Hsp90, and Cdc37 protein level were analyzed with western blotting using relevant antibodies. **(F)** SKBR3 cells were transiently transfected with control siRNA or Hsp90 siRNA for 48 h. Whole-cell lysates were analyzed with western blotting against HER2, Hsp90, and β-actin.

Most Hsp90 inhibitors, e.g.,17AAG, inhibit Hsp90 chaperone function by binding to Hsp90 N-terminal ATP pocket to prevent the maturation of Hsp90 client proteins [22]. Recombinant yeast NBD Hsp90 proteins were added with different concentrations of FW-04-806 or 17AAG to detect the effect of drugs on ATP-binding capacity of Hsp90. FW-04-806 (up to 40 μM) was unable to downregulate the amount of ATP-bound Hsp90, compared with the definite decrement caused by 17AAG, which suggested that FW-04-806 was not likely to decrease Hsp90 ATP-binding capacity (Figure 2A). In the colorimetric assay for inorganic phosphates to measure ATPase activity of Hsp90, FW-04-806 had little effect on the ATPase activity of Hsp90 and showed no dose-dependence, while the positive control 17AAG showed evident inhibition in a dose-dependent manner (Figure 2B).

According to computational docking, FW-04-806 inhibit Hsp90 chaperon capacity probably by disrupting the Hsp90 and co-chaperon Cdc37 complex, so we conducted the *in vitro* His-resin pull-down test by mixture recombinant Cdc37 and His tag Hsp90 protein with different concentrations of FW-4-806. The result showed FW-04-806 would hinder the interaction between Cdc37 and Hsp90 in a dose dependent manner (Figure 2C). Furthermore, we also immunoprecipitated Cdc37 and Hsp90 from whole-cell lysates of SKBr3, and analyzed the variations of bound Cdc37, Hsp90 and HER2 proteins. With the increment of FW-04-806, when Cdc37 was immunoprecipitated, the protein levels of bound Hsp90 and HER2 decreased. Vise verse, when Hsp90 was immunoprecipiated, the protein levels of bound Cdc37 and HER2 were decreased. These data indicated that the Hsp90/Cdc37/HER2 chaperone complex was damaged by FW-04-806 (Figure 2D).The effect of FW-04-806 on Hsp70, Hsp90, and Cdc37 was also tested using 17AAG as a positive control (Figure 2E). Hsp90 and Cdc37 protein levels showed no distinct change according to a drug concentration gradient, whereas Hsp70, a marker of Hsp90 inhibition, the protein level was greatly induced by treatment with 17AAG or FW-04-806. Furthermore, following depletion of Hsp90 protein by RNAi, FW-04-806 could not induce further HER2 protein degradation, suggested that Hsp90 protein was a direct target of FW-04-806 (Figure 2F).

### FW-04-806 decreases Hsp90 client protein levels and induces proteasome-dependent degradation

**Figure 3:**
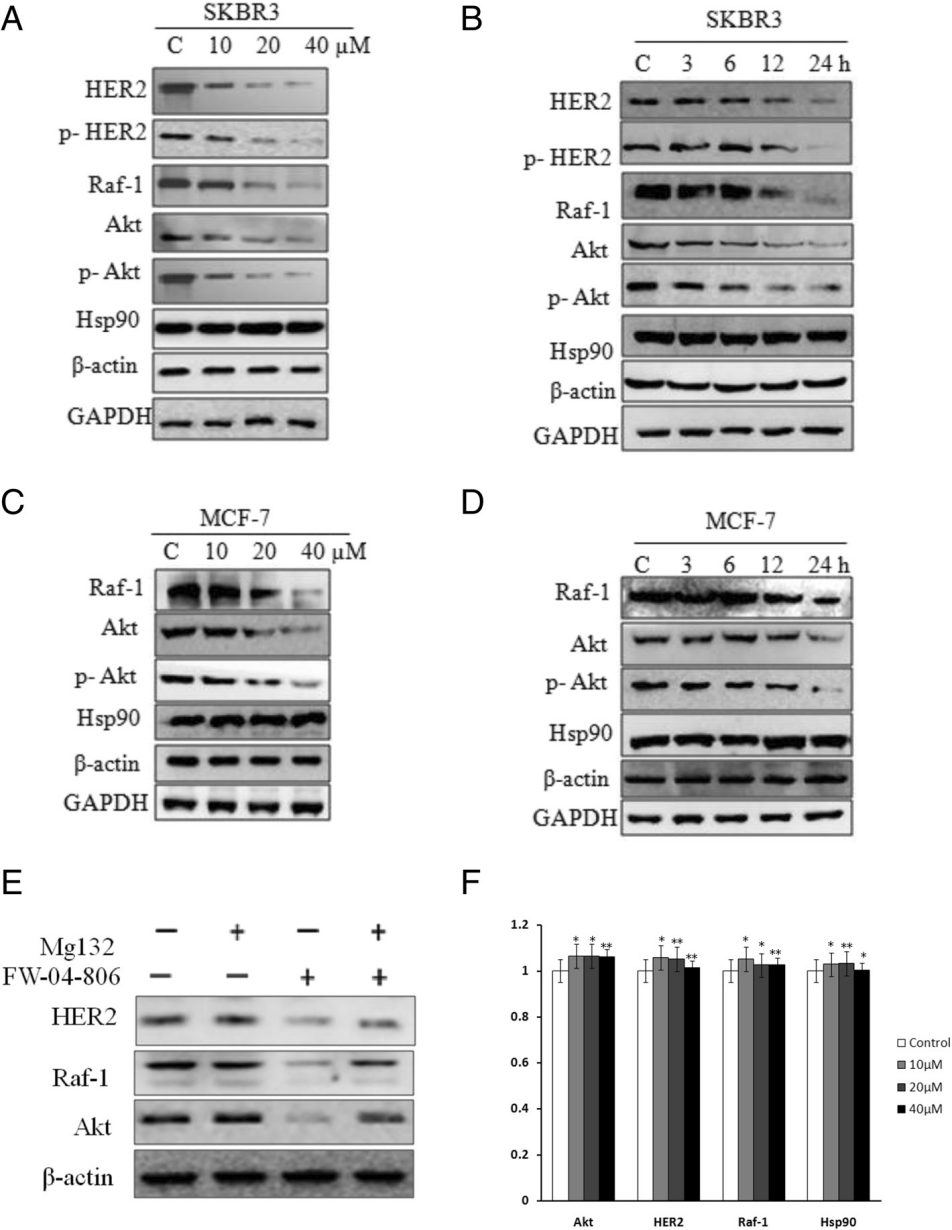
**FW-04-806 induces proteasome-dependent degradation and decreases Hsp90 client protein level. (A)** SKBR3 cells were treated by FW-04-806 at 10, 20, 40 μM for 24 h; HER2, phosphorylated HER2 (p-HER2), Raf-1, Akt, and phosphorylated Akt (p-Akt) protein levels were analyzed with western blotting. **(B)** SKBR3 cells were treated with 20 μM of FW-04-806 for 0, 3, 6, 12, or 24 h; HER2, p-HER2, Raf-1, Akt, and p-Akt protein levels were analyzed with western blot. **(C)** MCF-7 cells were treated by FW-04-806 at 10, 20, 40 μM for 24 h; western blot detected the protein expression of Raf-1, Akt, and p-Akt. **(D)** MCF-7 cells were treated with 20 μM FW-04-806 for 0, 3, 6, 12, or 24 h; western blot detected the protein expression of Raf-1, Akt, and p-Akt. **(E)** SKBR3 was pretreated with 1 μM of MG132 for 2 h in the presence or absence of 20 μM of FW-04-806 for an additional 12 h. Whole-cell lysates were subjected to western blot analysis using antibodies against HER2, Raf-1, Akt, and β-actin. **(F)** SKBR3 was treated with DMSO or FW-04-806 at 10, 20, 40 μM for 24 h. The total RNA was extracted for quantitative Real-time PCR of AKT, HER2, Raf-1 and Hsp90, using GAPDH as control. *P < 0.05: significant difference from control by analysis of variance; **P < 0.01: very significant difference from control by analysis of variance.

We tested the effect of the compound on the Hsp90 client proteins in breast cancer cells. SKBR3 and MCF-7 cells were treated with FW-04-806 at various concentrations and durations. FW-04-806 reduced the levels of the client proteins HER2, p-HER2, Raf-1, Akt, and p-Akt in a dose and time-dependent manner in SKBR3 cells (Figure 3A and B). The same tendency was observed in MCF-7 cells (Figure 3C and D), but no detectable protein level of HER2 was found in MCF-7 cell.In addition, degradation was completely blocked by treatment with the proteasome inhibitor MG132, indicating that the proteasome system was responsible for FW-04-806-induced client protein degradation (Figure 3E).Furthermore, we carried out quantitative real-time PCR to test the mRNA expression levels of Akt, HER2, Raf-1, Hsp90, using GAPDH as control. The result showed that FW-04-806 did not block the transcription, but directly acting through inhibition of Hsp90 (Figure 3F).

### FW-04-806 inhibits growth, induces cell cycle arrest, induces apoptosis, and downregulates the expression of anti-apoptotic proteins

**Figure 4:**
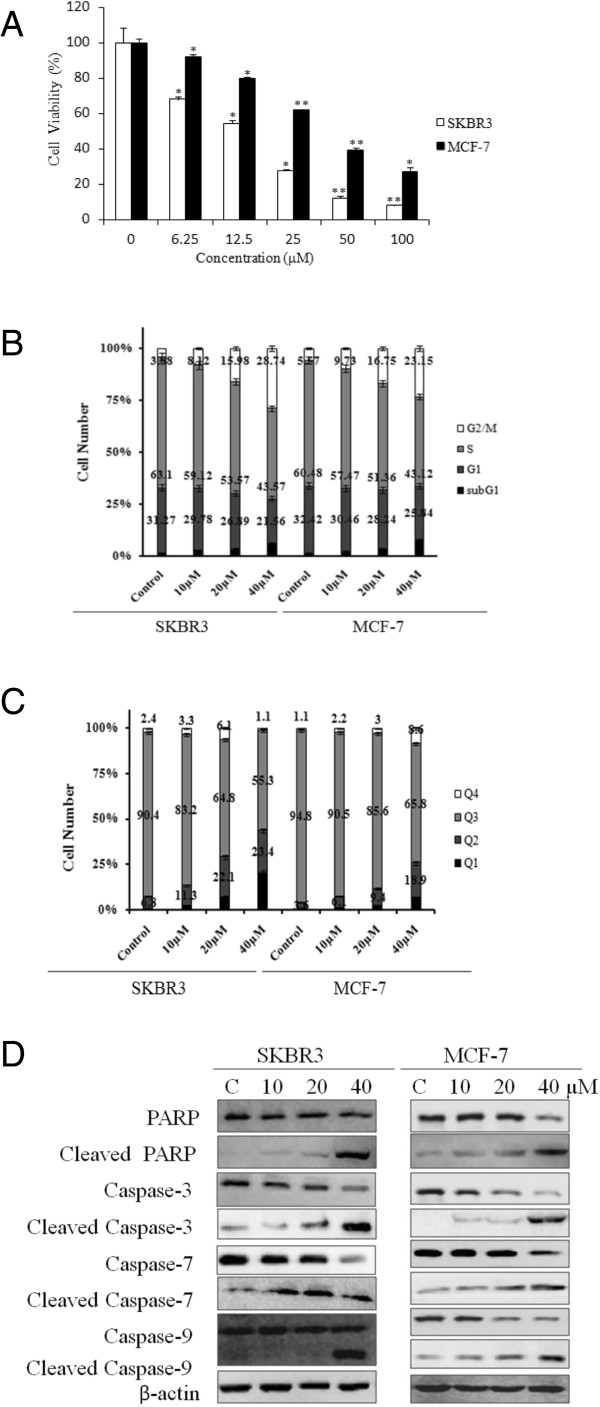
**FW-04-806 inhibits growth, induces cell cycle arrest, induces apoptosis, and downregulates the expression of anti-apoptotic proteins. (A)** SKBR3 and MCF-7 cells were grown for 48 h in the absence or presence of increasing concentrations of FW-04-806. Cell growth inhibition was measured with MTS and was expressed as a percentage of vehicle-treated control; results are presented as means ± SD of three independent experiments. *p < 0.05: significant difference from control by analysis of variance; **p < 0.01: very significant difference from control by analysis of variance. **(B)** SKBR3 and MCF-7 cells were treated with increasing doses of FW-04-806 or vehicle DMSO for 24 h. The cells were then fixed with 70% ethanol at −20°C overnight, incubated with RNase A at 37°C for 30 min, stained with propidium iodide for 10 min, and analyzed with flow cytometry. **(C)** SKBR3 and MCF-7 cells were treated with FW-04-806 for 24 h, and apoptotic cell death was detected by staining cells with an Annexin-V: FITC Apoptosis Detection Kit for analysis with flow cytometry. **(D)** SKBR3 and MCF-7 cells were treated with increasing doses of FW-04-806 for 24 h, and the apoptosis signal proteins were detected with western blot analysis using an Apoptosis Antibody Sampler Kit; β-actin was used as a loading control.

The effects of FW-04-806 on cell proliferation were assessed with an MTS assay. The proliferation of SKBR3 and MCF-7 cells was markedly inhibited by FW-04-806, with IC_50_ values of 12.11 and 39.44 μM, respectively (Figure 4A).Compared to vehicle-only treated controls, FW-04-806-treated cells displayed obvious arrest of cells in the G2/M phase after 24 h. The increase in the G2/M cell population was accompanied by a concomitant decrease in the population in the S and G0/G1 phases of the cell cycle (Figure 4B).SKBR3 and MCF-7 cells were treated with FW-04-806 for 24 h and analyzed for apoptotic cell death using an Annexin-V: FITC Apoptosis Detection Kit. The results revealed a dose-dependent induction of necrotic/late apoptotic cell death in both cell lines (Figure 4C).Caspases, a family of cysteine acid proteases, are central regulators of apoptosis. Western blot analysis revealed that FW-04-806 caused dose-dependent changes in the levels of apoptosis signal proteins. The initiator caspase 9, effector caspases (3 and 7), and the PARP precursor exhibited similar reductions, which were accompanied by increases in the levels of their cleaved fragments (Figure 4D). These data indicate that FW-04-806 induced apoptosis through caspase-dependent pathways in SKBR3 and MCF-7 cells.

### FW-04-806 inhibits the tumor growth of SKBR3 and MCF-7 tumor xenograft models

**Figure 5:**
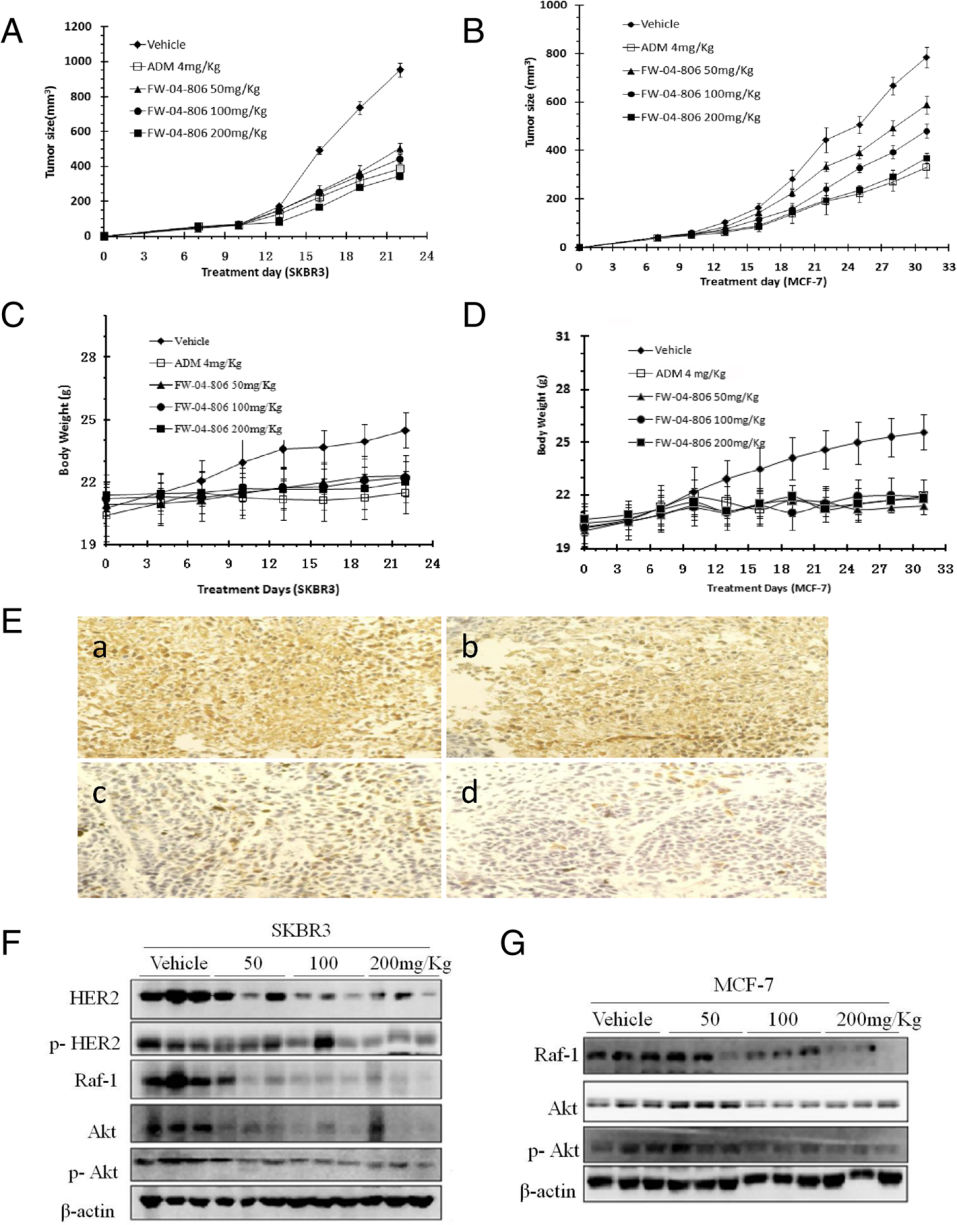
**FW-04-806 inhibits the tumor growth of SKBR3 and MCF-7 tumor xenograft models. (A** and **B)** SKBR3 and MCF-7 tumor xenograft nude mice were randomized into treatment groups (n = 6/group) and received FW-04-806 at doses of 50, 100, 200 mg/kg i.g., q3d (in a vehicle of 10% ethanol, 10% polyethylene glycol 400, and 10% Tween 80) or ADM at doses of 4 mg/kg i.p., q3d, until the day that the tumor sizes of the vehicle control groups reached approximately 1000 mm^3^. Tumors were weighed to evaluate the anticancer activity of FW-04-806. Data are presented as means ± SD (n = 6, P < 0.01). **(C** and **D)** Mouse body weight was measured twice a week. **(E)** HER2 expression in SKBR3 tumors. Picture a shows vehicle controls; picture b, c and d show 50, 100 and 200 mg/kg groups, respectively. **(F)** Tumor tissues excised from the SKBR3 xenograft mice were lysed; changes in the levels of in HER2, p-HER2, Raf-1, Akt, and p-Akt protein were tested. **(G)** Tumor tissues excised from the MCF-7 xenograft mice were lysed; western blot detected the changes in the levels of in Raf-1, Akt, and p-Akt protein expressions.

SKBR3 and MCF-7 human breast cancer xenografts were established to assess the chemotherapeutic potential of FW-04-806. The antitumor activity of FW-04-806 at three doses (50, 100, and 200 mg/kg per dose i.g., q3d) were determined. ADM (4 mg/kg per dose i.p., q3d) was used as a positive control. The results demonstrated that FW-04-806 inhibited tumor growth in the SKBR3 and MCF-7 xenograft models in a dose-dependent manner (Figure 5A and B). Compared with the vehicle group, the three increasing doses of FW-04-806 showed, respectively, inhibition of tumor growth at a rate of 39.1% (*P* = 0.009), 52.7% (*P* = 0.003), and 67.5% (*P* = 0.0007) in the SKBR3 cell line groups and 27.3% (*P* = 0.021), 39.8% (*P* = 0.004), 54.3% (*P* = 0.001) in the MCF-7 cell line groups. Notably, the antitumor activity of high-dose FW-04-806 (0.37 ± 0.04 g, 67.5%) was better than positive control group(0.39 ± 0.04 g, 64.9%, *P* = 0.0008).All animals survived FW-04-806 treatment without appreciable adverse effects in terms of body weight loss or other signs of toxicity during the treatment (Figure 5C and D). Liver and renal function was similar between FW-04-806-treated and control mice. Additionally, lung, liver, heart, and kidneys of mice showed no histological abnormalities at the end of drug treatment (data not shown). This outcome demonstrates that FW-04-806 was well tolerated.

Immunohistochemistry confirmed greater decreases in HER2 expression in the FW-04-806-treated groups compared with the vehicle groups in SKBR3 tumor xenografts; the reductions showed dose dependency (Figure 5E). The changes in HER2, p-HER2, Raf-1, Akt, and p-Akt protein levels were then checked in the excised tumor tissues. Western blotting results showed that high doses of FW-04-806 decreased the levels of p-HER2 and p-Akt in the same proportion as reductions in total HER2, Raf-1, and Akt in both SKBR3 and MCF7 model (Figure 5F and G). The results coincided with the western blot results *in vitro* (Figure 3).

## Discussion

Most Hsp90 inhibitors have been developed to inhibit Hsp90 chaperone function by binding to Hsp90 at the N-terminus and blocking the ATP/ADP pocket [22]. The antibiotics benzoquinone ansamycins, such as geldanamycin (GA) and its derivative 17-allyamino-geldanamycin (17AAG), were the first identified Hsp90 inhibitors [23]. The binding of GA in the N-terminal ATP pocket arrests the catalytic cycle of Hsp90 in the ADP-bound conformation, inactivating chaperone activity, which results in the ubiquitination and proteasomal degradation of client proteins [24-26]. Although GA and its derivatives have exhibited potent anticancer effects, severe hepatotoxicity has prevented clinical development [27]. This study showed that a natural product, FW-04-806, a novel Hsp90 inhibitor, inhibits Hsp90 function through binding the N-terminus of Hsp90 and blocking formation of the Hsp90-Cdc37 complex (Figures 1 and 2) in an ATP-binding independent manner, therefore the mechanism of action is clearly different from those classic Hsp90 inhibitors.

The wide-ranging functions of Hsp90 require a series of co-chaperones to drive the chaperone cycle to completion [22]. Therefore, affecting co-chaperone function by specifically targeting various co-chaperone/Hsp90 interactions may offer an alternative way to achieve the outcomes of direct Hsp90 inhibition [28,29]. Cdc37 is an essential co-chaperone and functions as an adaptor in the recruitment of client proteins, predominantly kinases such as HER2, epidermal growth factor receptor, non-receptor tyrosine kinases (Src), lymphocyte-specific protein tyrosine kinase, Raf-1, and CDK4, to Hsp90 [30-34]. The targeting of the Hsp90/Cdc37 interaction is a potential alternative to direct Hsp90 inhibition that may offer greater specificity and an improved side effect profile owing to the elevated expression of Cdc37 in cancer [5]. To date, only a few medicines were discovered to target Hsp90/Cdc37 interaction. Celastrol is a quinine methide triterpene extracted from *Tripterygium wilfordii*. It has recently been found to disrupt Hsp90/Cdc37 association, which results in the degradation of AKT and CDK4 and the induction of apoptosis in the pancreatic cell line Panc-1 [28]. But recent nuclear magnetic resonance (NMR) studies have suggested that celastrol binds to Cdc37 instead of the Hsp90 N-terminus domain [35,36]. Withaferin A (WA), a steroidal lactone extracted from *Withania somnifera*, disrupts the Hsp90/Cdc37 complex by binding to the C-terminus domain of Hsp90 and changing Hsp90 conformation to prevent Cdc37 binding [37,38]. Sulforaphane, a dietary component from broccoli sprouts, blocks Hsp90-Cdc37 complex by interacting with Ile amino acids residues of the N-terminal and middle domain of Hsp90 [19]. Our work here found a new medicine targeting Hsp90/Cdc37 interaction with new mechanism which is quite different with the medicines above.

We have showed that FW-04-806 is a Hsp90 inhibitor that directly binds to the N-terminus of Hsp90 and attenuates Hsp90/Cdc37 chaperone/co-chaperone interactions, leading to the degradation of multiple Hsp90 client proteins via the proteasome pathway, which may be the primary mechanism mediating the anticancer activities of FW-04-806. The antagonistic efficacy of FW-04-806 against human breast cancer lines has been investigated at both the molecular and cellular levels. It has been demonstrated that FW-04-806 inhibits the HER2-overexpressed and HER2-underexpressed breast cancer cell lines SKBR3 and MCF-7 in a dose and time-dependent manner with IC_50_ values of 12.11 and 39.44 μM, respectively. Moreover, it was shown that FW-04-806 arrests cell cycle progression and induces programmed cell death.

It has further been shown that FW-04-806 displays antitumor effects in an *in vivo* animal model as well as in the *in vitro* settings previously described. Studies were conducted to investigate the effect of FW-04-806 on tumors derived from cancer cell lines SKBR3 and MCF-7. High dose administration of FW-04-806 displayed an inhibitory effect on SKBR3-derived tumors was more preferable in both the antitumor activity and mouse body weight than that of ADM, one of the most widely used chemotherapy drugs. Importantly, we found that FW-04-806 displays a better antitumor effect in SKBR3 tumor xenograft model than in MCF-7. The result is consistent with cell proliferation assay and *in vitro* apoptosis assay applied for SKBR3 and MCF-7. As these cell lines are HER2-overexpressed and HER2-underexpressed respectively, and HER2 is among the most sensitive Hsp90 clients [39], we assume that FW-04-806 has a preferential inhibitory effect on HER2-overexpressed cancer cells. This assumption is now being tested on other cancer cell lines. Moreover, mice survived at the dose of 900 mg/kg in the acute toxicity test, and all xenografts mice had no appreciable adverse effects during the treatment. No histological abnormalities was found in lung, liver, heart, and kidneys of mice (Data not shown), suggested that FW-04-806 had a favorable toxicity profile.

## Conclusion

In conclusion, as a novel Hsp90 inhibitor, FW-04-806 binds to the N-terminal of Hsp90 and inhibits Hsp90/Cdc37 interaction, resulting in the disassociation of Hsp90/Cdc37/client complexes and the degradation of Hsp90 client proteins. FW-04-806 displays promising antitumor activity against breast cancer cells both *in vitro* and *in vivo*, especially for HER2-overexpressed breast cancer cells. Our observations provide a basis for the further development of Hsp90 or HER2 targeted therapy for patients with breast cancer.

## Competing interest

The authors declare that they have no competing interests.

## Authors’ contribution

WH and MY carried out the mechanism studies, participated equally in the experiments and drafted the manuscript. MY also conceived of the project, and participated in its design and coordination. LZ carried out the computational docking. QW and MZ participated in the western blot assay. JX and WZ conceived of the study, and participated in its design and coordination and helped to draft the manuscript. All authors read and approved the final manuscript.
